# Network Features of the Mammalian Circadian Clock

**DOI:** 10.1371/journal.pbio.1000052

**Published:** 2009-03-10

**Authors:** Julie E Baggs, Tom S Price, Luciano DiTacchio, Satchidananda Panda, Garret A FitzGerald, John B Hogenesch

**Affiliations:** 1 Department of Pharmacology and the Institute for Translational Medicine and Therapeutics, University of Pennsylvania School of Medicine, Philadelphia, Pennsylvania, United States of America; 2 Medical Research Council, Social Genetic and Developmental Psychiatry (SGDP) Centre, Institute of Psychiatry, King's College London, London, England, United Kingdom; 3 Regulatory Biology, Salk Institute for Biological Studies, La Jolla, California, United States of America; University of Geneva, Switzerland

## Abstract

The mammalian circadian clock is a cell-autonomous system that drives oscillations in behavior and physiology in anticipation of daily environmental change. To assess the robustness of a human molecular clock, we systematically depleted known clock components and observed that circadian oscillations are maintained over a wide range of disruptions. We developed a novel strategy termed Gene Dosage Network Analysis (GDNA) in which small interfering RNA (siRNA)-induced dose-dependent changes in gene expression were used to build gene association networks consistent with known biochemical constraints. The use of multiple doses powered the analysis to uncover several novel network features of the circadian clock, including proportional responses and signal propagation through interacting genetic modules. We also observed several examples where a gene is up-regulated following knockdown of its paralog, suggesting the clock network utilizes active compensatory mechanisms rather than simple redundancy to confer robustness and maintain function. We propose that these network features act in concert as a genetic buffering system to maintain clock function in the face of genetic and environmental perturbation.

## Author Summary

The circadian clock is the biological clock found throughout the body that coordinates the timing of molecular and cellular processes on a 24-hour rhythm. It is composed of numerous transcription factors that feed back and control their own expression. To explore how the clock functions in the face of genetic perturbations, we disrupted its function by knocking down gene expression of known clock genes in a dose-dependent fashion. We measured the expression of clock genes following knockdown and constructed perturbation-based network models to describe, visualize, and mine the results. We reported several novel network features, such as signal propagation through interacting genetic modules and proportional responses whereby levels of expression are altered commensurately with changing levels of the gene. We also observed several examples where a gene is up-regulated following knockdown of its paralog, suggesting the clock network utilizes active compensatory mechanisms rather than simple redundancy to confer robustness and maintain function. We propose that the network features we observe act in concert as a genetic buffering system to maintain clock function in the face of genetic and environmental perturbation.

## Abstract 3

How does the circadian clock maintain function in the face of genetic perturbation? The authors construct gene dosage perturbation networks and uncover several underlying principles contributing to genetic buffering of the clock.

## Introduction

The mammalian circadian clock is a cell-autonomous system that drives oscillations in behavior and physiology in anticipation of daily environmental change. These oscillations manifest through interactions of the central pacemaker in the suprachiasmatic nucleus (SCN) of the hypothalamus with local circadian clocks present in most mammalian tissues [1]. Many clock components have been identified that act to generate circadian transcriptional oscillations through a regulatory system comprised of negative feedback loops [2,3]. Studies in mouse models indicate that relatively few molecular perturbations of clock components (e.g., knockout or mutant animals) lead to complete loss of oscillator function as assessed by locomotor activity or circadian gene expression in isolated tissues [2]. This suggests that the molecular clockwork constitutes a regulatory module that is phenotypically robust, i.e., resistant to and/or buffered against genetic perturbations such as gene loss, deletion, or mutation [4]. Consistent with these findings, it often takes deletion of multiple factors (often gene paralogs) to disrupt behavioral and/or cellular rhythms (summarized in Table 1).

**Table 1:**
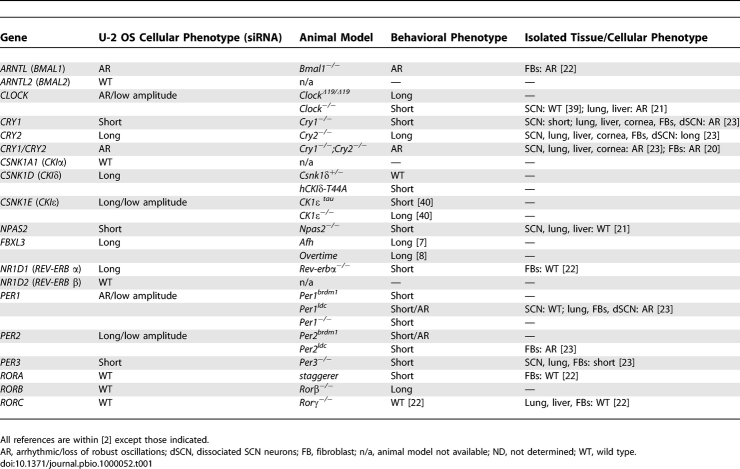
Effects of Gene Knockout or Knockdown in Animal Models and U-2 OS Cells

The majority of identified clock components function as transcriptional activators or repressors. These proteins, along with other components that modulate protein stability and nuclear translocation, create two interlocking transcriptional feedback loops [2,3]. At the core of the oscillator are two transcriptional activators, CLOCK and BMAL1, which heterodimerize and bind to E-box elements in the promoters of target genes, including two families of transcriptional repressors, the PERIOD (*Per1*, *Per2*, and *Per3*), and CRYPTOCHROME (*Cry1* and *Cry2*) proteins, as well as in other genes that regulate outputs mediated by the clock. Upon accumulation in the cytoplasm, PER and CRY proteins enter the nucleus and inhibit their own expression by repressing CLOCK/BMAL1-mediated transcription [2,3]. Multiple proteins regulate stability and/or nuclear accumulation of clock components, including casein kinase I family members (CSNK1D and CSNK1E) and the F-box and leucine-rich repeat protein 3 (FBXL3) [5–8]. A second stabilizing feedback loop interlocks with the primary loop and regulates *Bmal1* expression positively by retinoic acid receptor–related orphan receptors (ROR) transcriptional activators and negatively by REV-ERB transcriptional repressors through binding to retinoic acid-related orphan receptor elements (ROREs) in the *Bmal1* promoter [9–13]. Paralogs of several clock genes exist, such as *Bmal2* and *Npas2* (paralogs of *Bmal1* and *Clock*, respectively), which display similar biochemical functions yet may regulate unique target genes due to differential spatial expression [14–17].

Cellular models of clock function such as immortalized cell lines (e.g., NIH3T3 cells), mouse embryonic fibroblasts (MEFs), and dissociated cells derived from the SCN, recapitulate robust oscillator function yet are free from neural, humoral, and behavioral cues that influence the clock in the intact organism [18,19]. Many important properties of circadian behavior are maintained in these isolated autonomous oscillators. For example, MEFs derived from knockout mice of the transcriptional repressors *Cry1* and *Cry2* maintain defects in period length consistent with the short- and long-period locomotor behavior rhythms observed in the respective knockout mice [20]. These cellular models are ideal for genetic perturbation experiments, as clock gene dosage can be altered over a wide range of concentrations. The consequences of these perturbations can be measured using kinetic imaging, biochemistry, and gene expression analysis.

Here, we present comprehensive genetic perturbation analysis of a new model of the autonomous human circadian clock. Using this system, we analyzed the functional consequences of decreasing expression of individual and combinations of circadian clock components in a dose-dependent fashion using RNA interference (RNAi). Effects on cellular oscillations were monitored using kinetic imaging, and changes in gene expression of other clock components were assessed by quantitative real-time PCR (RT-PCR). Using these data and applying biochemical constraints, we constructed gene interaction networks to describe, visualize, and mine the perturbation-induced changes for emerging network features. Disruption of gene function using previous methods, e.g., comparing knockout versus wild-type animals, lacked the statistical power necessary for uncovering such changes due to a lower number of gene doses (zero, one, or two copies). Using this new method, we uncovered several novel features of the circadian clock, including proportional response of gene expression, where levels of expression are altered actively and in a proportional fashion with respect to the gene being knocked down. By measuring the responses of most clock factors following perturbation of a single gene, we also observed signal propagation through interacting activator and repressor modules. Furthermore, our method uncovered unidirectional paralog compensation among several clock gene repressors, providing the first example to our knowledge of multiple paralogs regulated in this fashion in a single pathway. We propose that the features we uncovered provide mechanisms to buffer the clock against genetic perturbation, and ultimately contribute to the extraordinary robustness of the oscillator.

## Results and Discussion

### A Robust Circadian Oscillator Is Resistant to Perturbation of Most Components

**Figure 1:**
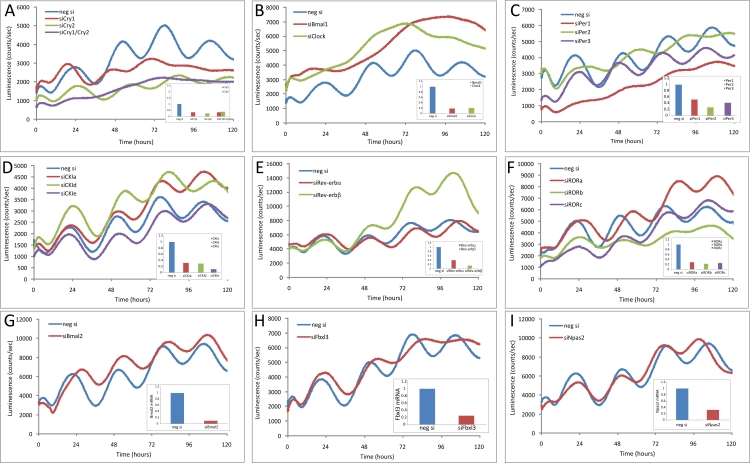
Functional Effects on Oscillations Following Knockdown of Circadian Clock Components in U-2 OS Cells U-2 OS cells were transfected with pools of four to five siRNAs for each gene, and oscillations in *Bmal1*-luciferase were measured for 5 d following synchronization with dexamethasone. Knockdown of each component was validated by quantitative RT-PCR from RNA isolated from a replicate sample. The relative amount of expression of each gene compared to the negative siRNA control sample is shown (insets). Results are representative of three independent biological replicates.

To investigate the network features of the circadian clock that underlie its robustness, we used small interfering RNAs (siRNAs) to knockdown clock components in immortalized human osteosarcoma cells (U-2 OS cells) that are amenable to both RNAi and quantitative kinetic imaging. Following depletion, mRNA levels of the targeted components were measured using quantitative RT-PCR. Cells stably expressing luciferase under the control of the *Bmal1* promoter displayed robust oscillations in bioluminescence with a period length of approximately 24 h for more than 6 d in culture after synchronization with dexamethasone (Figure S1). We compared our knockdown experiments to behavioral phenotypes and cellular oscillations observed in mice lacking *Cry1* and *Cry2*. As expected, knockdown of *Cry1* in our model led to a short-period phenotype, whereas knockdown of *Cry2* produced a long-period rhythm when compared to controls (Figure 1A). Similar to behavioral and cellular phenotypes from double-knockout mice, cotransfection of *Cry1* and *Cry2* siRNAs in U-2 OS cells led to complete loss of circadian oscillations (Figure 1A). This recapitulation of in vivo circadian behaviors provided initial validation of this model system.

The remaining clock components (*Bmal1*, *Clock*, *Per1*, *Per2*, *Per3*, *CKIa*, *CKId*, *CKIe*, *Rev-erb alpha*, *Rev-erb beta*, *RORa*, *RORb*, *RORc*, *Bmal2*, *Fbxl3*, and *Npas2*) were similarly depleted, and *Bmal1*-luciferase oscillations were monitored in kinetic assays to explore the requirement of other clock genes for oscillator function (Figure 1, Table 1). Remarkably, most treatments had either no effect or caused only small changes in period length. The depletion of only three genes, however—*Clock*, *Bmal1*, and *Per1*—disrupted robust circadian oscillations in bioluminescence (Figure 1B and 1C). In all treatments, we observed 60%–80% decrease in gene expression of the targeted gene, indicating that the absence of severe phenotypes for other clock genes was not due to ineffective knockdown (Figure 1). In general, the observed changes in oscillator function were consistent with the behavioral and/or tissue level rhythms seen in knockout mice (Table 1). For example, lack of robust oscillations following knockdown of *Clock*, *Bmal1*, or *Per1* was also observed in cultured peripheral tissues and/or fibroblasts derived from these respective knockout mice (Figure 1B and 1C) [21–23]. Furthermore, the phenotypes we observed in knockdown of *Cry1* (short, Figure 1A), *Cry2* (long, Figure 1A), *Per3* (short, Figure 1C), *RORa* (wild type, Figure 1F), or *RORc* (wild type, Figure 1F) are consistent with published findings in most cellular and tissue models [20,22,23]. However, we observed a few differences from the reported phenotypes of knockout mice, namely that period length changes in human U-2 OS cells following knockdown of *Npas2* (short, Figure 1I), *Rev-erb alpha* (long, Figure 1E), or *Per2* (long, Figure 1C) were different than the behavioral and/or tissue-level phenotypes reported in knockout mice, suggesting that the composition of the circadian clock varies in different tissue types or mammalian species. Collectively, these studies along with work from animal models highlight the robust nature of the circadian clock to genetic perturbation, as few *single* clock gene knockouts or knockdowns disrupt oscillator function.

### Generation of Genetic Networks to Describe Perturbation Effects

To investigate the network features that contribute to robust oscillator function in response to genetic perturbation, we developed a method called Gene Dosage Network Analysis (GDNA). Reasoning that the statistical power to analyze these studies was constrained by the number of perturbations, we based GDNA on the ability of RNAi to generate multiple levels of knockdown at finer intervals than is possible using traditional genetics approaches (i.e., from wild-type levels to approximately 10% residual expression). Following dose-dependent depletion of each clock component, mRNA levels of other clock components were measured using quantitative PCR. Starting with the perturbed component, nonparametric Pearson correlations of relative gene expression levels were calculated and the shortest paths, or edges, were drawn between the perturbed gene and all other components in the network consistent with the biochemical nature of the perturbed component (i.e., targets of activators go *down* when activators are depleted; targets of repressors go *up* when repressors are depleted). To attribute signal propagation through the network, this process was repeated with first-order perturbed components (i.e., genes directly connected to the perturbed gene) until no further edges could be drawn. Unlike sequence analysis of gene regulatory regions, which predicts occupancy of transcription factors, or chromatin immunoprecipitation, which shows occupancy and predicts function, the GDNA procedure results in a genetic network based on the *observed functional* consequences of perturbation. It is important to note that although GDNA networks are consistent with many biochemical observations, they are genetic networks and not intended to be biochemically mechanistic.

**Figure 2:**
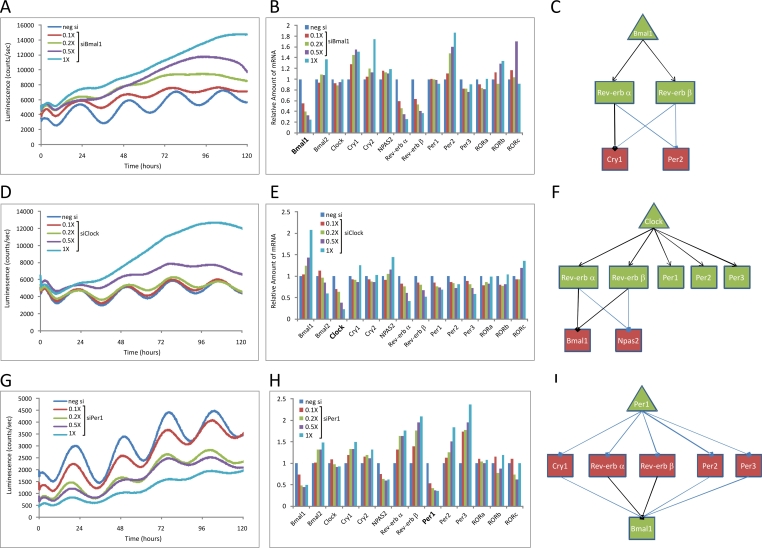
GDNA Following Genetic Perturbation of *Bmal1*, *Clock*, and *Per1* (A, D, and G) Oscillations in *Bmal1*-luciferase were measured from cells transfected with increasing amounts of siRNAs targeting *Bmal1*, *Clock*, or *Per1*. (B, E, and H) RNA was isolated from replicate samples collected at time 0 (before synchronization) and expression of clock genes was determined by quantitative real time PCR. (C, F, and I) GDNAs were generated for *siBmal1* (C), *siClock* (F), and *siPer1*(I) using expression data from each knockdown condition. Edges between genes were determined using nonparametric Pearson correlation (*p*-value < 0.10) and biochemical constraints, and gene expression changes are denoted as increases (red) and decreases (green). The gene being depleted is located at the top of network, with first order responses below as restricted by biochemistry, i.e. genes that decrease when *Clock* and *Bmal1* activators are decreased (C and F) and genes that are increased with the *Per1* repressor is decreased (I). Second-order responses are defined as those with correlated edges with the first-order responders. Black lines indicated published edges, and blue lines denote unpublished relationships.

We used GDNA to help characterize network features of the clock during conditions that generated loss of robust oscillator function, i.e., knockdown of *Clock*, *Bmal1*, and *Per1*. Knockdown of either of the obligate heterodimers *Bmal1* or *Clock* led to similar defects in function, with a dose-dependent loss of circadian rhythms and increasing levels of the *Bmal1*-luciferase reporter (Figure 2A and 2D), whereas knockdown of *Per1* also led to loss of circadian rhythms, but with decreasing levels of the *Bmal1*-luciferase reporter (Figure 2G). mRNA levels of clock genes were measured in these samples, and we observed dose-dependent decreases in the target gene (*Bmal1*, *Clock*, or *Per1*) as well as dose-dependent changes in other circadian genes (Figure 2B, 2E, and 2H). Despite all of these perturbations leading to loss of robust oscillations, no two perturbation conditions produced the same effects on gene expression. Knockdown of *Bmal1* led to a decrease in *Rev-erb alpha* and *Rev-erb beta* and an increase in *Per2* and *Cry1* (Figure 2B). Knockdown of *Clock* led to decreases in *Rev-erb alpha*, *Rev-erb beta*, *Per1*, *Per2*, and *Per3*, and increases in *Bmal1* and *Npas2* (Figure 2E), whereas knockdown of *Per1* led to increases in *Rev-erb alpha*, *Rev-erb beta*, *Cry1*, *Per2*, and *Per3* and decreased *Bmal1* (Figure 2H). The dose-dependent changes in gene expression we observed in unsynchronized cells (Figure 2) were also observed throughout the circadian cycle in synchronized, oscillating cells (Figure S2). Gene Dosage Networks (GDNs) were constructed from these data by drawing edges between the gene targeted by siRNA knockdown and genes that change in a manner consistent with the biochemical function of the target gene. For example, since BMAL1 is a transcriptional activator, its target genes must go down when *Bmal1* levels are decreased; therefore, in the *siBmal1* condition, direct edges are drawn between *Bmal1* and both *Rev-erb alpha* and *Rev-erb beta* (Figure 2C). The observed increases in *Cry1* and *Per2* following knockdown of *Bmal1* could not occur directly by the BMAL1 transactivator, however, and therefore are likely to occur through signal propagation by the transcriptional repressors *Rev-erb alpha* and *Rev-erb beta*. As a result, we have drawn edges between the *Rev-erb*s and *Cry1* and *Per2* (Figure 2C). GDNs were constructed in a similar fashion following perturbation of *Clock* (Figure 2F) and *Per1* (Figure 2I). This analysis revealed edges consistent with published observations in addition to several novel edges, such as dose-dependent decreases in mRNA expression of *Rev-erb alpha* [22,24] and *Rev-erb beta* in response to loss of function of *Bmal1* or *Clock* (Figure 2C and 2F). Thus, this approach captures known aspects of oscillator function while providing a framework to generate novel hypotheses regarding clock network function.

### Network Features: Proportional Response

**Figure 3:**
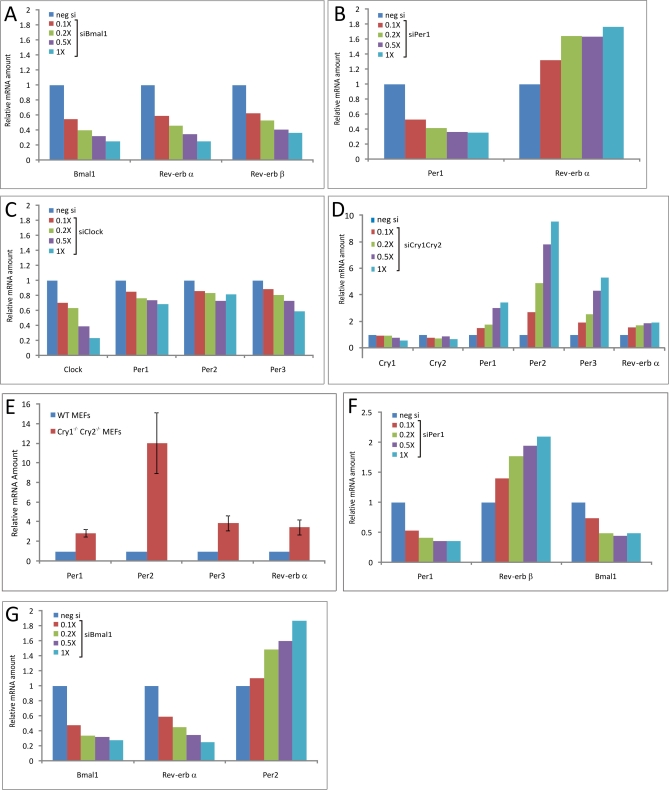
Signal Propagation of Proportional Gene Expression Changes Is Observed upon Dose- Dependent Knockdown of Clock Components (A–C) Examples of linear, proportional gene expression changes following knockdown of *Bmal1* (A), *Per1* (B), and *Clock* (C). (D and E) Disproportional responses are observed in *Per2* gene expression following knockdown of *Cry1* and *Cry2* (D), and similar changes are observed in MEFs derived from *Cry1/Cry2* double-knockout mice (E). (F) Signal propagation following knockdown of *Per1* through a Repressor/Repressor module where knockdown of *Per1* leads to increase of *Rev-erb*s, which in turn causes a decrease in *Bmal1* expression. (G) An Activator/Repressor module relays the signal following knockdown of *Bmal1*, which leads to a decrease in *Rev-erb alpha* and a subsequent increase in *Per2*.

One goal of systems biology is to identify governing properties that underlie the function of biological networks. One feature of the circadian regulatory network uncovered by GDNA is the proportional responses in downstream gene expression following siRNA-induced perturbation. These proportional responses can be described using the simple linear equation, *y* = *b*_o_ + *b*_1_ *x*, where *b*_o_ is the baseline expression of the gene, *b*_1_ is the slope of the response, *x* is the expression level of the gene targeted for knockdown, and *y* is the expression level of the downstream gene. This is best exemplified using the results generated under *Bmal1*, *Clock*, *Per1* (Figure 2), and *Cry1/2* (Figure S3) knockdown conditions. Following dose-dependent knockdown of *Bmal1*, we observed linear proportional decreases in *Rev-erb alpha* and *Rev-erb beta* mRNA levels (Pearson correlations of *r* = 0.99 and *r* = 1.00, and slopes of *b*_1_ = 0.97 and *b*_1_ = 0.85, respectively) (Figure 3A). This is most easily explained by direct activation of the *Rev-erb* genes by Bmal1, which has been previously described [11,13], and suggests that *Bmal1* is a strong contributor to *Rev-erb* expression. In the previous example of *Rev-erb* response to knockdown of *Bmal1*, the relationship is considered strictly proportional because the slope *b*_1_ is close to 1. Whereas targets of activators respond proportionally to their knockdown, targets of repressors respond in an inverse and proportional fashion. An example of an inverse linear proportional change is the response of *Rev-erb alpha* following depletion of *Per1*, where *Rev-erb* levels increase in a linear, but inversely proportional, fashion with respect to the decrease in *Per1* (*b*_1_ = −0.9) (Figure 3B). However, not all of the relationships are strictly proportional. For example, the *Per* genes respond to *Clock* knockdown in a linear and proportional, but fractional, fashion, i.e., although the *Per* genes decrease when *Clock* levels fall, the relationship is best described by a fractional slope (*b*_1_ = 0.4 [Per1], *b*_1_ = 0.3 [Per2], and *b*_1_ = 0.5 [Per3]) (Figure 3C). Finally, we see disproportional responses (with the absolute value of the slope greater than 1) of *Per2* and *Rorc* genes following depletion of Cryptochromes. An approximately 50% knockdown of *Cry1/Cry2* results in a 10-fold induction in *Per2* and *Rorc* mRNA expression levels (slope is not calculated because of the double-knockdown condition) (Figures 3D and S3F). We validated these disproportional changes in gene expression in MEFs derived from wild-type and *Cry* double-knockout mice (Figure 3E). While the vast majority of genetic responses to perturbation are proportional, with a slope close to ±1, we observed disproportional responses only in the *Cryptochrome* depletion condition. These disproportional responses are likely to be specific to the biochemical activity of cryptochromes, and not simply because both paralogs were depleted, as these responses were also observed in the *Cry1* single-knockdown condition (when comparing *Cry1* and *Per2*, *b*_1_ = −4.2) (Figure S3B). Thus, this principle of proportionality takes several forms in the circadian network, including direct and inverse, strict, fractional, and disproportional.

This principle of proportional response has been suggested in sporadic reports in the literature over the last two decades [25]. For example, using a knockout mouse model to compare two doses of gene depletion, Rudnicki et al. [26] noted a 1.8-fold induction of *Myf5* in *MyoD1* heterozygote animals, and a 3.6-fold proportional induction of *Myf5* in *MyoD1* homozygous null animals [25,26]. In the case of the circadian network, we provide dozens of examples showing that proportional response is the rule, rather than the exception, of the clock gene network. This principle of proportionality has attractive implications for quantitative network deconstruction and modeling.

### Network Features: Signal Propagation through Interacting Modules

Another feature we observed in the circadian network is signal propagation through interacting genetic modules of activators and repressors. The GDNs provide a framework to uncover these biological relay mechanisms through which modules communicate changes in gene expression. We observed combinations of proportional response modules that relay and propagate perturbation signaling through the clock network. For example, in the *Per1* perturbation network, we observed signal propagation through Repressor/Repressor modules resulting in a strictly proportional decrease in the level of *Bmal1* (Figure 3F). When *Per1* is depleted, the *Rev-erb*s increase (which is likely due to the repressive function of PER1 on E-box sequences in the *Rev-erb* promoters), then that signal propagates to the target of the *Rev-erb* repressors, *Bmal1*, and is observed as a strictly proportional decrease in its expression (Figure 3F). In another example, we observed an Activator/Repressor relay following knockdown of *Bmal1*. By knocking down the activator *Bmal1*, we see a decrease in the *Bmal1* target gene *Rev-erb alpha* and an increase in *Per2*, which is likely a direct or indirect target of *Rev-erb* repression (Figure 3G). We propose that the clock network combines these activator and repressor modules with various forms of proportionality to construct relays that generate complex gene expression responses to single gene perturbations.

### Network Features: Paralog Compensation

**Figure 4:**
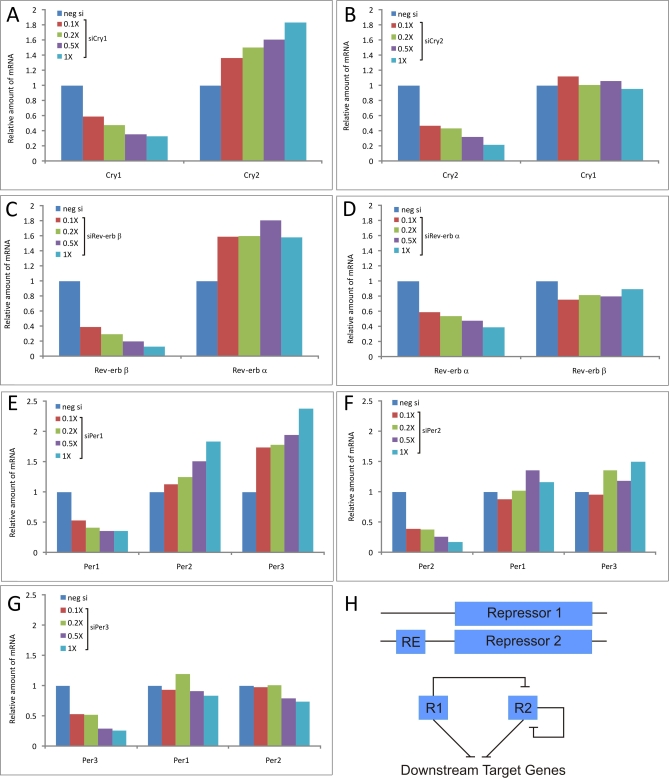
Unidirectional Paralog Compensation in the Circadian Repressors *Cry1*, *Rev-erb beta*, and *Per1* (A, C, and E) U-2 OS cells were transfected with doses of siRNA as indicated, and gene expression was measured from samples collected 48 h after transfection. Knockdown of *Cry1* (A), *Rev-erb beta* (C), and *Per1* (E) leads to increased expression of the gene paralogs *Cry2*, *Rev-erb alpha*, and *Per2* and *Per3*, respectively. (B, D, F, and G) The response is unidirectional, as a decrease of *Cry2* (B), *Rev-erb alpha* (D), *Per2* (F), or *Per3* (G) does not result in an increase of respective gene paralogs. (H) Transcriptional repressors could directly regulate the expression of their gene paralog through response elements (RE) in upstream regulatory regions (H) (modified from [25]). In the case of the circadian clock network, we propose direct regulation of *Cry1*, *Per1*, and *Rev-erb beta* (Repressor 1) by *Cry2*, *Per2/3*, or *Rev-erb alpha* (Repressor 2), respectively.

Another prominent feature of clock network organization, paralog compensation, was revealed using GDNA. Following knockdown of several transcriptional repressors, we observed the up-regulation of gene paralog(s), usually transcriptional repressors, through an active mechanism by which gene function is replaced following knockdown. Following a decrease in *Cry1* expression, *Cry2* mRNA levels increased (Figure 4A), *Rev-erb alpha* mRNA was induced when *Rev-erb beta* was depleted (Figure 4C), and knockdown of *Per1* led to a subsequent increase in both *Per2* and *Per3* (Figure 4E). This active method of gene compensation is distinct from redundancy, where one component is sufficient to sustain function. In addition, a high resolution time course of gene expression from synchronized, oscillating U-2 OS cells confirms that the unidirectionality of these changes cannot be explained by the phase of mRNA expression of these gene paralogs in the circadian clock (unpublished data; GEO accession GSE13949). Finally, the finite number of gene doses in knockout animals (a maximum of three: wild type, heterozygous, and homozygous) may explain why paralog compensation has not been widely observed in circadian network organization (aside from the increase in *Npas2* following knockout of *Clock* in [24]) or other signal transduction pathways.

The phenomenon of paralog compensation (or genetic backup) was first described on the genome-wide scale in yeast [27]. In addition, other examples have been reported, including several genes that provide compensatory backup during vertebrate development (summarized in [25]). Network motifs that generate this genetic backup have been termed responsive backup circuits [25]. Interestingly, most paralogous gene pairs with demonstrated backup function exhibit unidirectional changes, i.e., only two gene pairs of the 16 examples summarized in Kafri et al. [25] can regulate each other in a bidirectional fashion. This unidirectional mode of regulation is also observed in the circadian network (e.g., *Cry2* knockdown has no effect on *Cry1* expression) (Figure 4B, 4D, 4F, and 4G). Several mechanisms have been proposed to describe these responsive backup circuits [25]. Because the clock components that show paralog compensation are transcriptional repressors, we propose a responsive backup circuit structure with direct negative regulation of these clock factors (*Cry2*, *Per2/3*, and *Rev-erb alpha*) by their paralog (*Cry1*, *Per1*, and *Rev-erb bet*a, respectively) (Figure 4H). The role of gene paralogs in maintaining robustness of circadian oscillations is well established in animal models (e.g., *Cry1/Cry2* or *Per1/Per2*) [2].

### Network Features: a Role for Nonparalogous Compensation

**Figure 5:**
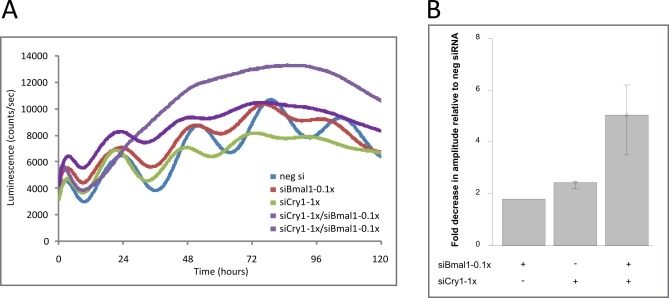
Compensation in Nonparalogous Genes May Contribute to Oscillator Robustness (A) U-2 OS cells transfected with siRNAs targeting *Bmal1* or *Cry1*, individually or in combination as indicated, were synchronized, and luminescence levels were measured over 5 d. Data are representative of three independent biological replicates. Note that two examples are shown for the *siCry1/siBmal1* combination knockdown. (B) Median and ranges of amplitude following single or combinatoric perturbation. The amplitude of the circadian signal was estimated using continuous wavelet decomposition and averaged across three replicates in three independent experiments. The median and range of the fold changes relative to the negative siRNA control are plotted. Both individual siRNA treatments are significantly down-regulated relative to the negative siRNA controls (*p* < 0.05, Mann-Whitney test), and the *siCry1/siBmal1* double knockdown is also significantly down-regulated relative to *siCry1* (*p* < 0.05, Mann-Whitney test).

Paralog compensation may not solely provide the compensatory backup sufficient to maintain the oscillator in a functionally operational state. For example, Liu et al. [23] showed that loss of *Per1* or *Cry1* led to arrhythmicity in explanted tissues, suggesting that compensatory backup of these components is not sufficient to sustain oscillations. However, there are clearly differences between our system and the model in Liu et al., as we observe a functional oscillator in U-2 OS cells following knockdown of *Cry1*. This may suggest that the networks are wired differently depending on the tissue and/or species, or that genetic knockout and knockdown produce variation in phenotype. Because most compensatory gene expression changes we observe in the U-2 OS network occur in nonparalogous genes (Figure 2B, 2E, and 2H), we propose that other gene expression changes may assist in conferring robustness to the oscillator. We set out to test this by preventing such changes following knockdown of *Bmal1* using combinations of siRNAs under conditions that normally support high amplitude oscillations (50% reduction in *Bmal1*). We hypothesized that the compensatory increase in *Cry1* following knockdown of *Bmal1* contributed to oscillator function, and indeed, we observed a decrease in amplitude, an increase in arrhythmic cells, and more variability among replicate samples with double knockdown of *Bmal1* and *Cry1* relative to knockdown of each individual component alone (Figure 5). Surprisingly, we did not observe this combinatorial defect in function upon cotreatment with siRNAs targeting *Bmal1* and *Per2* (unpublished data), suggesting that perhaps some, but not all, compensatory gene expression changes contribute to oscillator robustness. Therefore, we propose extending the concept of responsive backup circuits to encompass and define these nonparalogous, compensatory interactions to facilitate hypothesis generation and computational modeling while defining edges that confer robustness to the oscillator.

### Network Features: Genetic Buffering

We propose that the features we have described—proportional response, signal propagation through activator and repressor modules, and compensation through gene expression changes—act in concert to maintain clock components within functional biochemical ranges. Gene expression changes occurring under numerous perturbations may promote this genetic buffering effect. For example, knockdown of *Clock* led to dose-dependent decreases in its repressors, *Per1*, *Per2*, and *Per3*, and increases in its heterodimeric partner *Bmal1* and paralog *Npas2* (Figure 2E and 2F). Thus, upon depletion of *Clock*, compensatory changes occur to balance mRNA levels of activators (*Bmal1* and *Npas2*) and repressors (*Pers*), and maintain them within operational ranges (tolerant of nearly 70% knockdown in *Clock* message; Figure 2D and 2E). Similar changes occur upon genetic perturbation of *Per1*. Lower *Per1* levels results in an increase in the E-box–driven *Rev-erb alpha* and *Rev-erb beta* genes and a subsequent decrease in *Bmal1*, likely through direct RORE-mediated repression of *Bmal1* by REV-ERB [11]. The transcriptional network that emerges upon *Per1* perturbation enables Bmal1, a target of the PER/CRY repression complex, to remain in balance with one of its repressors, Per1 (Figure 2H and 2I), thus providing a method to maintain components at operational levels with respect to one another. We therefore propose that the network features of the clock described above act in concert to promote genetic buffering.

### Conclusions

Diploid organisms display remarkable resistance to genetic change (promoter mutations, loss of function alleles, copy number variation including gene deletion, allelic silencing, etc.). For example, only 1% of the more than 4,000 mouse knockout lines that have been created are heterozygous lethal (JAX mice database, http://www.jax.org). Conversely, approximately 20% of all homozygous knockout mice are embryonic lethal (http://www.jax.org). Therefore, the sum of all pathways, networks, and processes required to take a single-celled organism through development into a viable postnatal mouse are tolerant of 50% reduction in dosage of the vast majority of genes. Furthermore, gene loss is by no means the most prevalent cause of variability in gene dosage. In multicellular organisms, most genes are expressed in a tissue-specific fashion and often vary dramatically in their expression depending on cell type, tissue, or structure. Many conventionally defined pathways function throughout the organism (e.g., cell cycle, circadian clock, NFkB, Ap1, cAMP pathways) even though the relative expression of key pathway components varies across tissues [28]. Therefore, many biological pathways have evolved genetic networks that function to account for this variance and maintain function in the face of genetic variation [29]. In fact, this property has been hypothesized as necessary for evolution: a hardened genetic network intolerant of changes in gene dosage would be less likely to accrue genetic mutations that could later be selected upon under pressure [30].

How is this robustness to genetic perturbation achieved? Large-scale analysis of the haploid Saccharomyces cerevisiae suggests that active, systems-level mechanisms are in place to achieve robustness [27]. We hypothesized that these active mechanisms must be at play in pervasive biological networks/pathways, such as the circadian clock, that function throughout the body of mammals. To explore these mechanisms, we devised a novel strategy termed Gene Dosage Network Analysis that combines dose-dependent knockdowns with functional and biochemical analysis. This analysis revealed strikingly simple principles of proportional response, signal propagation through activator and repressor modules, and paralog compensation that manifest in the highly buffered clock network. Proportionality took several forms—fractional and strict—and the disproportional responses seen in knockout of the *Cry* genes, which act as potent repressors. Upon perturbation, interacting activator and repressor modules produced signal propagation through key relay points to achieve complex and unexpected gene expression patterns. We also found several straightforward examples of paralog compensation, which is enabled by responsive backup circuits and acts to restore gene activity following knockdown of circadian repressors. These features, which we suggest interact to produce genetic buffering, may in part explain the prevalence of tissue-specific circadian clocks, despite tissue-specific variance in expression of clock components.

Previous investigations of functional systems have tended to contrast “passive” structural features (such as the existence of paralogs) that lend robustness to genetic knockout through redundancy of function with “active” rerouting of metabolic pathways in response to graded environmental exposures [31,32]. We contend that our investigation of graded *genetic* perturbation provides evidence that responsive backup circuits play a role in maintaining dynamic features of systems behavior, a function that has been hypothesized, but not empirically demonstrated [25]. Importantly, these results demonstrate that no two perturbations produced the same changes in the network architecture; although there were common features in the resultant GDNA networks, each was distinct (Figure S4). Such flexibility in the network interactions among genes has also been suggested in functional gene networks that influence coordination in flies [33]. This property allows us to successfully model functional and biochemical consequences of *previously measured* perturbations, but casts significant doubt as to the value and predictive nature of a single integrated model or our ability to model combinatory effects that have not been experimentally explored.

In addition, these data are consistent with a new model in which the circadian clock is composed of a genetic buffering system intermingled with a biochemical oscillator (Figure S4). This model agrees with several recent findings indicating that transcriptional oscillations of key clock components are dispensable for circadian function [22,34]. We propose that many of these oscillations are secondary and a consequence of network wiring. For example, loss of both *Rev-erb alpha* and *Rev-erb beta* still results in a functional oscillator even though *Bmal1* oscillations are disrupted (unpublished data and [22]), indicating that this secondary feedback loop is not required for oscillator function and therefore may function to promote stability and robustness. Furthermore, these data support the idea that in addition to biochemical modeling of negative feedback loops, the molecular clock should be modeled at the network level [35,36]. We propose extending the GDNA approach to investigate clock gene changes over time, in combination, and in different cellular and tissue contexts to learn more about network structure and function. Furthermore, by expanding the GDNA to encompass genome-wide measurements following perturbation, we can explore for novel components and network features. Finally, although the exact circuitry may differ by cell or tissue type, these general principles may be utilized in all circadian clock systems and indeed, in other biological networks, to maintain function in the face of genetic, tissue-specific, and environmental perturbation.

## Materials and Methods

### siRNA sequence information.

The target sequences of circadian gene siRNAs are supplied in Table S1. All siRNAs are from Qiagen or Applied Biosystems.

### siRNA transfections and kinetic measurement of luminescence.

U-2 OS cells stably transfected with *Bmal1*-luciferase reporter were seeded at 1.5 × 10^5^ cells in 35-mm dishes in DMEM medium containing 10% fetal bovine serum (FBS) and 0.1 mM nonessential amino acids (NEAA) (Invitrogen). Cells were transfected with siRNAs (12 pmol [1×] to 1.2 pmol [0.1×], as indicated) using Lipofectamine 2000 transfection reagent (Invitrogen) following the manufacturer's instructions. A negative control siRNA (AllStars Negative control siRNA; Qiagen) was used to ensure equal molar amounts of siRNA was added in each condition. In the case of double knockdowns, a maximum of 15 pmol was transfected per well (with 1× corresponding to 7.5 pmol of each siRNA, and 0.1× corresponding to 0.75 pmol of each siRNA). Two days posttransfection, the medium was changed to phenol red–free DMEM containing 10% FBS, NEAA, 1× penicillin/streptomycin/glutamine (PSG) (Invitrogen), 10 mM HEPES (Invitrogen), 0.1 mM luciferin (Promega), and 0.1 μM dexamethasone (Sigma-Aldrich), and the dishes were covered with sterile glass coverslips, sealed with sterile vacuum grease, and placed into the Lumicycle luminometer (Actimetrics). Luminescence levels were measured every 10 min for 5 d or more.

### Maintenance and plating of MEFs.

Wild-type MEFs and MEFs derived from *Cry1^−/−^;Cry2^−/−^* knockout mice were maintained in DMEM supplemented with 10% FBS/1× NEAA/1× PSG. RNA was extracted (see methods below) from cells plated in 35-mm dishes 2 d after cells were seeded at 1 × 10^5^ cells/dish.

### Isolation of RNA and gene expression assays.

RNA was isolated using both Trizol (Invitrogen) and the RNeasy Mini kit (Qiagen) in which the homogenization and phase separation was performed following the manufacturer's instructions for Trizol, and the aqueous phase was column purified following the RNeasy Mini kit instructions. Reverse transcription of 0.5–1 μg of RNA was performed using the High Capacity cDNA Reverse Transcription kit (Applied Biosystems) and quantitative RT-PCR was performed using TaqMan gene expression assays (Applied Biosystems) and iTaq Supermix with Rox master mix (BioRad) according to the manufacturers' instructions. Individual TaqMan gene expression assays for ARNTL (TaqMan gene expression probe Hs00154147_m1), ARNTL2 (Hs 00368068_m1), CLOCK (Hs00231857_m1), CRY1 (Hs00172734_m1), CRY2 (Hs00391360_m1), CSNK1A1 (Hs00793391_m1), CSNK1D (Hs01020332_m1), CSNK1E (Hs00266431_m1), NPAS2 (Hs00231212_m1), NR1D1 (Hs00253876_m1; Mm00520708_m1), NR1D2 (Hs00233309_m1), PER1 (Hs00242988_m1; Mm00501813_m1), PER2 (Hs00256144_m1; Mm00478113_m1), PER3 (Hs00536545_m1; Mm00478120_m1), RORA (Hs00536545_m1), RORB (Hs00199445_m1), and RORC (Hs01076112_m1) were used to detect expression levels in U-2 OS cells and MEFs on a 7800HT TaqMan machine (Applied Biosystems). Endogenous controls against GAPDH (human) and ubiquitin C (mouse; Mm01201237_m1) were used for normalization. All data were analyzed using RQ Manager v1.2 (Applied Biosystems).

### Calculation of period length and amplitude.

We characterized the circadian oscillation in the cell luminescence data using wavelet decomposition techniques that simultaneously detrend, denoise, and describe the data without making parametric assumptions about trends in the frequency or amplitude of the component signals. The time series data were projected into a time-frequency matrix using continuous Morlet wavelet transformation. The modal frequencies were identified and reconstructed using the “crazy climbers” algorithm [37]. Summaries of the period and amplitude of the reconstructed circadian signal were calculated in the interval excluding the “cone of influence” at the edges of the time-frequency space. All calculations were performed on a Dell desktop PC running R for Windows version 2.7.0 (http://www.R-project.org) using the clockwave package version 1.0–0 [38].

### Generation of GDNA networks.

Dose-dependent knockdown of each component was accompanied by mRNA expression measurements of itself and other components in the circadian clock network using quantitative RT-PCR. Each expression profile was normalized to its negative siRNA transfection control. Nonparametric Pearson analysis was performed using the perturbed component's mRNA expression profile as the first vector and mRNA expression of other components as each subsequent vector, thus generating the “true” Pearson correlation. To estimate *p*-value, the expression measurements for each component of the vector were shuffled 1,000 times without replacement, and the true Pearson value was ranked in the distribution of these shuffled values. Edges were drawn when the Pearson correlation had a *p*-value of less than 0.10 and the target of the perturbed component behaved in a manner consistent with its established biochemical properties (we defined *Bmal1*, *Bmal2*, *Clock*, *Npas2*, and *RORs* as activators and *Cry1*, *Cry2*, *Per1*, *Per2*, *Per3*, and *Rev-erb*s as repressors). For example, beginning with the perturbed gene, we drew a line or edge between that component and all other genes that had a significant Pearson score, and gene changes occurred in the correct direction depending on whether the perturbed component was an activator or repressor. For example, for activators, edges were drawn to all components that had positive and significant Pearson scores, whereas for repressors, all target components had negative and significant Pearson scores. Following the first round of this analysis, additional rounds were performed from the second-order genes until all significant edges (with Pearson scores consistent with biochemical properties) were exhausted. Components targeted by siRNA are indicated by a green triangle indicating the knockdown of that gene. Genes that responded to this perturbation by reduction of their mRNA are indicated in green boxes, while induced genes are indicated by red boxes. Previously described edges in the literature (e.g., *Bmal1* regulation of *Rev-erb alpha* and *Rev-erb beta*) are indicated by a black line, and novel edges are indicated in blue. Where multiple genes could influence the expression of another component, all plausible edges were drawn. Of note, these networks require direct or indirect connectivity to the perturbed component. There are cases where significant Pearson scores were possible, but no connection to the perturbed component could be drawn. In these cases, these satellite networks were disregarded. Finally, these networks are meant as plausible representations of genetic networks consistent with biochemical properties and are not intended to be biochemically mechanistic.

## Supporting Information

Figure S1 U-2 OS Cells Contain a Robust Circadian Clock Luminescence levels were measured from U-2 OS cells stably transfected with a *Bmal1*-luciferase reporter for 6 d, following synchronization with dexamethasone at time 0.
(164 KB JPG)

Figure S2 Gene Expression Changes Resulting from siRNA Knockdown Are Conserved in Unsynchronized and Oscillating Cell Populations U-2 OS cells were transfected with doses of siRNAs targeting *Bmal1*, and samples were collected from unsynchronized cells (B) or at 6-h intervals in oscillating cells (C–H), as indicated by arrows in (A). Expression of *Bmal1*, *Per2*, and *Rev-erb alpha* was measured using quantitative RT-PCR. Expression of each gene was normalized to the negative siRNA control sample at each time point.
(1.17 MB JPG)

Figure S3 Knockdown of Cryptochromes Disrupts Circadian Oscillator Function (A, C, and E) U-2 OS cells were transfected with varying doses of siRNAs against *Cry1* (A), *Cry2* (C), or both *Cry1* and *Cry2* (E). Cells were synchronized by dexamethasone treatment and oscillations were measured for 5 d.
(B, D, and F) Gene expression for circadian clock components was assessed in unsynchronized samples 48 h posttransfection.
(1.30 MB JPG)

Figure S4 Responses to Perturbation of the Circadian Gene “Network” Under normal conditions, gene interactions (solid black lines) among genes (dark blue circles) are maintained, as illustrated in this hypothetical network of gene interactions in the circadian clock. However, under specific perturbation conditions, for example decreases in levels of *Bmal1*, *Clock*, or *Per1*, the genetic network responds with conditional interactions (indicated by dashed lines), and can bypass or amplify canonical interactions. Although each perturbation condition leads to a similar phenotype—the loss of robust oscillations—the molecular alterations that resulted were unique to each perturbation. (Figure modified from Greenspan, 2001 [29].)
(537 KB JPG)

Table S1 Target Sequences of siRNAs. (78 KB DOC)

## Abbreviations

GDNA: Gene Dosage Network Analysis
MEF: mouse embryonic fibroblast
RNAi: RNA interference
ROR: retinoic acid receptor-related orphan receptors
RT-PCR: real-time PCR, SCN, suprachiasmatic nucleus
siRNA: small interfering RNA
U-2 OS: human osteosarcoma cell line
