# Supplementary material for: Network Features of the Mammalian Circadian Clock
Source: PLoS Biol. 2009 Mar 10;7(3):e1000052. doi: 10.1371/journal.pbio.1000052 (PMC2653556; doi:10.1371/journal.pbio.1000052)
Supplement: Supplementary file 1 [file pbio.1000052.st001.doc]

**Supplemental Table 1. Target Sequences of siRNAs.**
